# miR-137 acts as a tumor suppressor via inhibiting CXCL12 in human glioblastoma

**DOI:** 10.18632/oncotarget.20589

**Published:** 2017-08-24

**Authors:** Dehua Li, Wei Shan, Yan Fang, Pan Wang, Jicheng Li

**Affiliations:** ^1^ Institute of Cell Biology, Zhejiang University, Hangzhou, 310031, China; ^2^ Department of Human Anatomy, Histology and Embryology, School of Medicine, Zhejiang University, Hangzhou, 310031, China; ^3^ Department of Anatomy, College of Basic Medical Sciences, Jinzhou Medical University, Jinzhou, 121000, China

**Keywords:** miR-137, CXCL12, GBM, suppressor

## Abstract

Up to date, miR-137 has been demonstrated as a tumor suppressor in many kinds of human malignancies. In the present study, we conducted transfection, western blot and RT-PCR to explore the role of miR-137 in the development of human glioblastoma (GBM). Here, we found that miR-137 expression was obviously down-regulated in GBM tissues and cells rather than matched non-tumor tissues and NHA cells. However, the expression of C-X-C motif ligand 12 (CXCL12) mRNA and protein were up-regulated in GBM tissues and cells. *In vitro*, miR-137 mimics inhibited GBM cell proliferation, migration and invasion, and the 3′-untranslated regions (3′-UTR) of CXCL12 were a direct target of miR-137. In addition, miR-137 mimics also inhibited the expression of EGFR, Bcl-2 and MMP2/9 proteins, but increased the expression of Bax protein. Notably, CXCL12 over-expression attenuated miR-137-inhibited cell proliferation and invasion, while CXCL12 siRNAs promoted miR-137 inhibition effects. *In vivo*, miR-137 mimics also suppressed tumor growth in nude mice xenograft model. In conclusion, miR-137 serves as a tumor suppressor by inhibition of CXCL12 in human GBM. Thus, miR-137-CXCL12 can be recommended as a useful and effective target for treatment of GBM.

## INTRODUCTION

To date, human astrocytomas have been classified into four grades by The World Health Organization (WHO) based on tumor type [1–4]. Grade IV astrocytoma is also called as glioblastoma multiforme (GBM), which is the most common and malignant type of gliomas, and accounts for more than 60% of astrocytomas [5, 6]. Since the histopathological grade of gliomas can not reflect the pathological molecular mechanisms that drives the tumor biology, it is essential to seek a better diagnostic, prognostic and therapeutic target for GBM patients.

MicroRNAs (miRNAs) are a kind of non-coding RNAs, which contains small endogenous 19–23 nucleotides [7]. In many kinds of human malignancies, miRNAs serves as oncogenes or tumor suppressor genes to regulate translation processes or mediate degradation of mRNAs according to the features of their targets [8]. Previous reports suggested that miR-137 expression is reduced and suggested as a prognosis biomarker in many kinds of tumors, involving lung cancer, colorectal cancer [9], and oral squamous cell carcinoma [10]. In addition, CXCL12 has been reported to be involved into the development of glioblastoma [11–13]. However, the role of miR-137/CXCL12 in human GBM is still unknown.

In the present work, we explored and detected the expression of miR-137 and CXCL12, and then we investigated the role of miR-137 and CXCL12 on GBM cell proliferation, migration and invasion using *in vitro* assays, and elucidate the anticancer effects of miR-137. We further evaluated whether CXCL12 is an objective target of miR-137, which would be a potential target for treatment of human glioblastoma.

## RESULTS

### The expression of miR-137 and CXCL12 in GBM tissues

**Figure 1:**
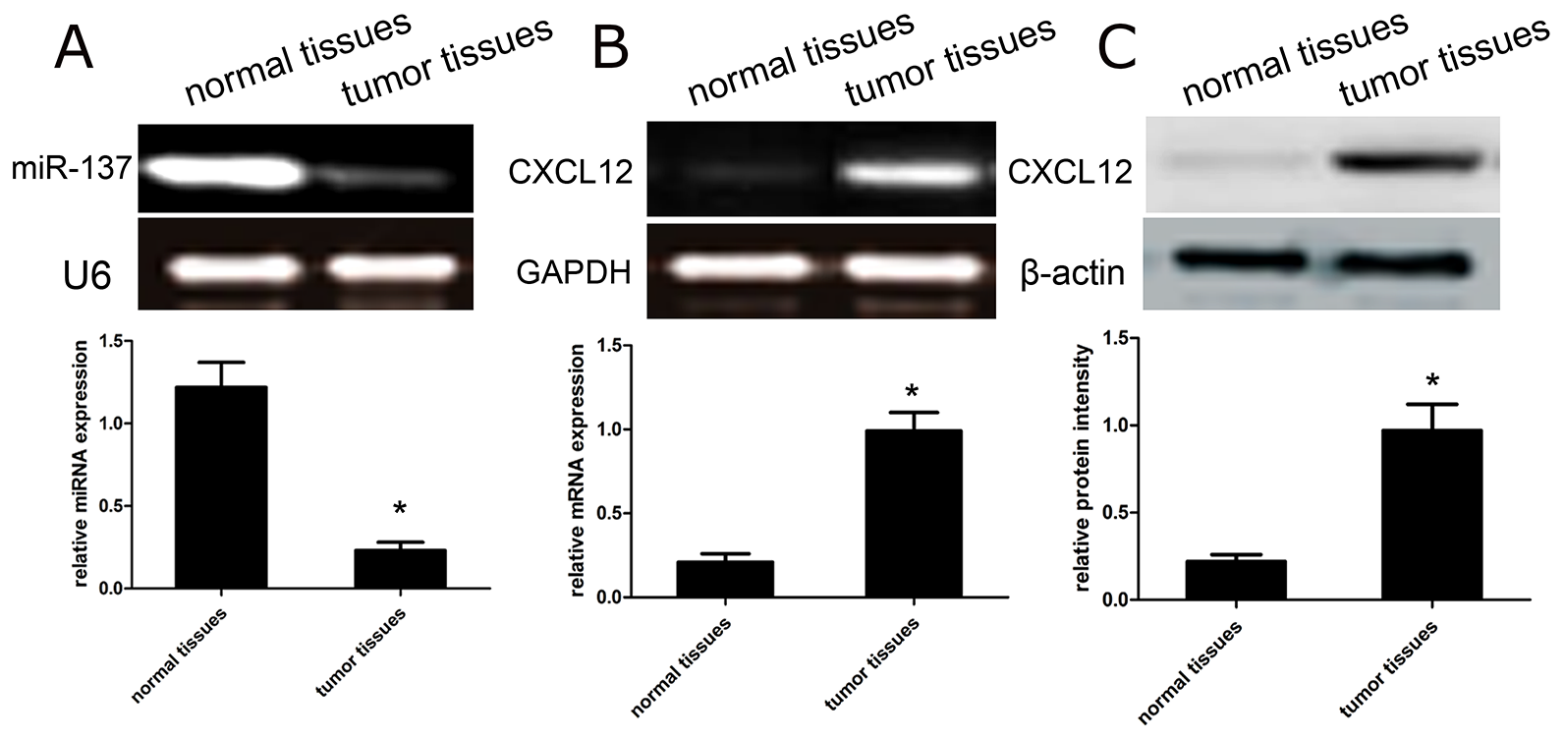
The expression profile of miR-137 and CXCL12 in GBM tissues **(A-B)** The RT-PCR analysis of miR-137 and CXCL12 mRNA expression were conducted in tumor tissues and matched non-tumor tissues. Quantification analysis was defined as the relative density of miR-137 and CXCL12 mRNA to U6 and GAPDH, respectively. U6 or GAPDH was used as an internal control. Results shown are the mean ± SD of repeated independent experiments. ^*^p<0.01, compared with normal tissues, one-way ANOVA. **(C)** The expression of CXCL12 protein was examined in tumor tissues and matched non-tumor tissues using western blot. The average CXCL12 protein expression was normalized to β-actin. Results shown are the mean ± SD of 3 repeated independent experiments. ^*^p<0.01, compared with normal tissues, one-way ANOVA.

To figure out the role and significance of miR-137 and CXCL12 in the development of GBM, we firstly detected the expression of miR-137 and CXCL12 in 50 cases of tumor tissues and paired adjacent non-tumor tissues. We found the expression of miR-137 was obviously decreased in tumor tissues than that in paired non-tumor tissues (p<0.01; Figure 1A). However, the expression of CXCL12 mRNA was obviously increased in tumor tissues than that in non-tumor tissues (p<0.01) (Figure 1B). Furthermore, the expression of CXCL12 protein was found to be significantly higher in tumors than that in non-tumor tissues (p<0.01) (Figure 1C). Overall, the optical density of CXCL12 mRNA and protein in 50 cases of tissues is 0.85 ± 0.11, and 1.22 ± 0.16, respectively, compared with those in paired non-tumor tissues (0.29 ± 0.06, and 0.35 ± 0.06, respectively).

### The expression of miR-137 and CXCL12 in GBM cell lines

**Figure 2:**
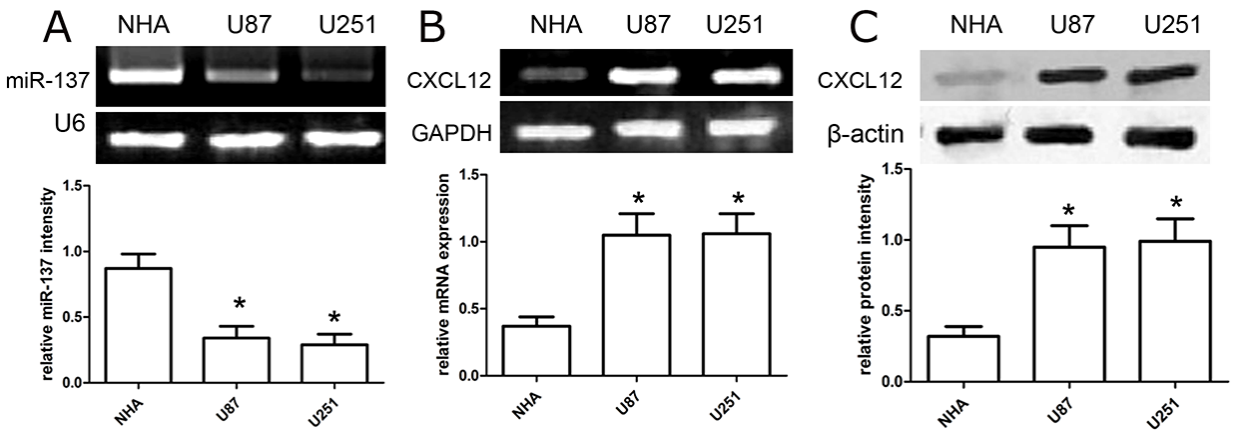
The expression profile of miR-137 and CXCL12 in GBM cell lines **(A-B)** RT-PCR analysis of miR-137 and CXCL12 expression in glioblastoma U87 and U251 cell lines. Quantification analysis was defined as the relative density of miR-137 and CXCL12 mRNA to U6 and GAPDH respectively. U6 or GAPDH was used as an internal control. Results shown are the mean ± SD of repeated independent experiments. ^*^p<0.01, compared with NHA cells, one-way ANOVA. **(C)** The expression of CXCL12 protein was examined in GBM cell lines U87 and U251 using western blot. The CXCL12 expression was normalized to β-actin expression. Results shown are the mean ± SD of 3 repeated independent experiments. ^*^p<0.01, compared with NHA cells, one-way ANOVA.

Based on results above, we evaluated the expression of miR-137 and CXCL12 in U87 and U251 cell lines. In the present study, the expression detection of miR-137 and CXCL12 was subjected to qRT-PCR analysis. We found that the expression of miR-137 was significantly decreased in U87 and U251 cells, while the expression of miR-137 was obviously increased in normal NHA cells (p<0.01; Figure 2A). On the other hand, the expression of CXCL12 mRNA was obviously up-regulated in U87 and U251 cells instead of normal NHA cells (p<0.01) (Figure 2B). In addition, the expression of CXCL12 protein was also increased in U87 and U251 cells as compared with normal NHA cells (p<0.01) (Figure 2C).

### Effects of miR-137 on GBM cell proliferation

**Figure 3:**
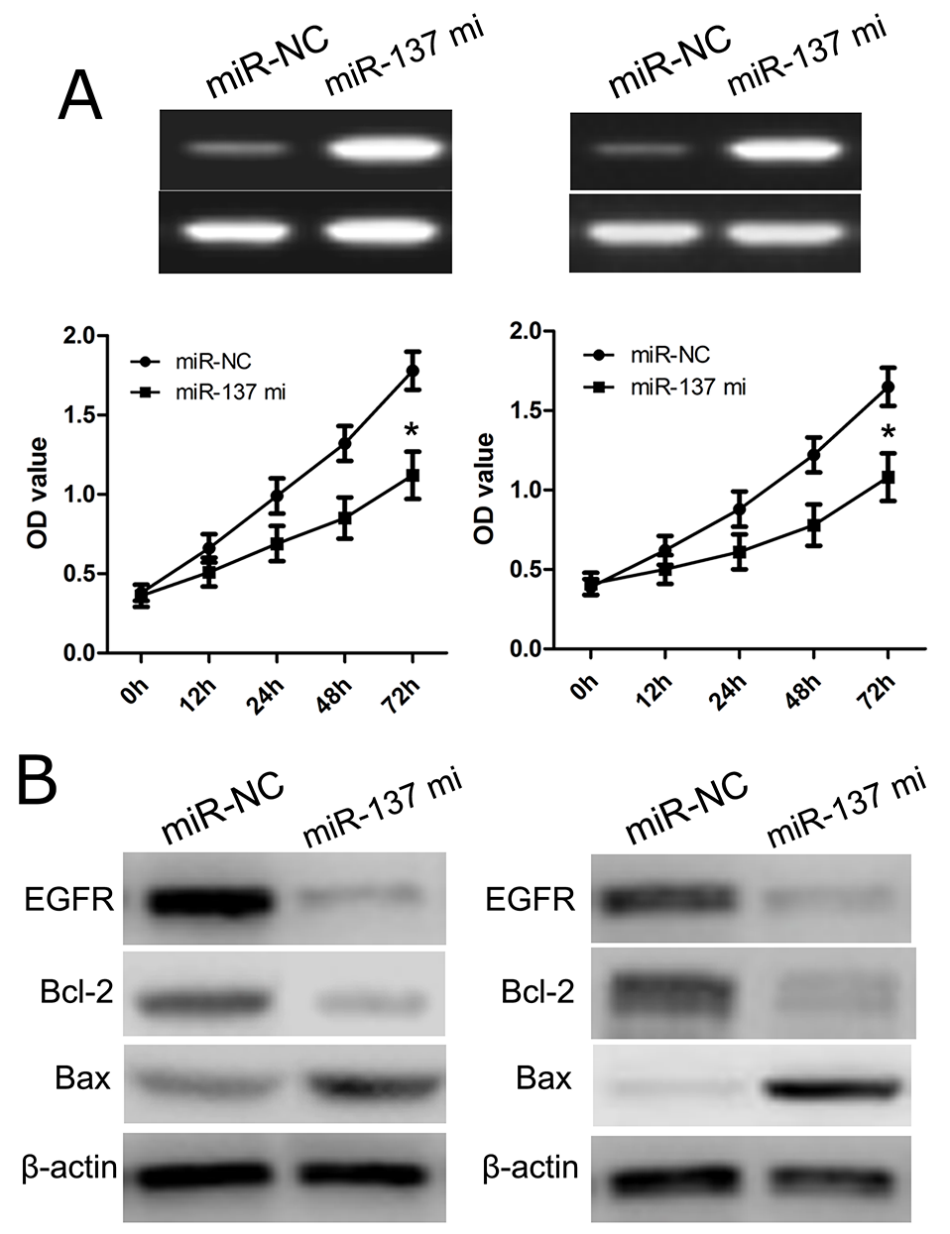
miR-137 inhibits glioblastoma cell proliferation **(A)** Cells were transfected with miR-137 mimics and identified by RT-PCR. Cell proliferation was measured using a CCK-8 assay. U87 and U251 cells were transfected with miR-137 mimics or scramble control. **(B)** Relative EGFR, Bcl-2 and Bax expression in U87 and U251 cells was measured after the cells were transfected with miR-137 mimics or NC miRNA using western blot. Results shown are the mean ± SD of 3 repeated independent experiments. ^*^p<0.01, compared with miR-NC, one-way ANOVA.

In this work, we transfected U87 and U251 cells with miR-137 mimics or NC miRNA (miR-NC), and then used CCK-8 assay analysis to evaluate cell proliferation. We found that miR-137 mimics effectively reduced the number of viable cells of U87 and U251 cells, while cells in miR-NC group were not affected (p<0.01) (Figure 3A). This result suggested that cell proliferation was obviously inhibited owing to transfection of miR-137 mimics. Furthermore, we applied western blot to evaluate cell proliferation-related molecules, including EGFR, Bcl-2 and Bax. We found that the expression of EGFR and Bcl-2 protein was significantly decreased in U87 and U251 cells transfected with miR-137, while the expression of EGFR and Bcl-2 protein in miR-NC group were not affected (Figure 3B). Meanwhile, the expression of Bax protein was also increased in U87 and U251 cells transfected with miR-137 mimics as compared with miR-NC group (Figure 3B). These findings indicated that miR-137 expression affected cell proliferation in the development of GBM.

### Effects of miR-137 on GBM cell migration and invasion

**Figure 4:**
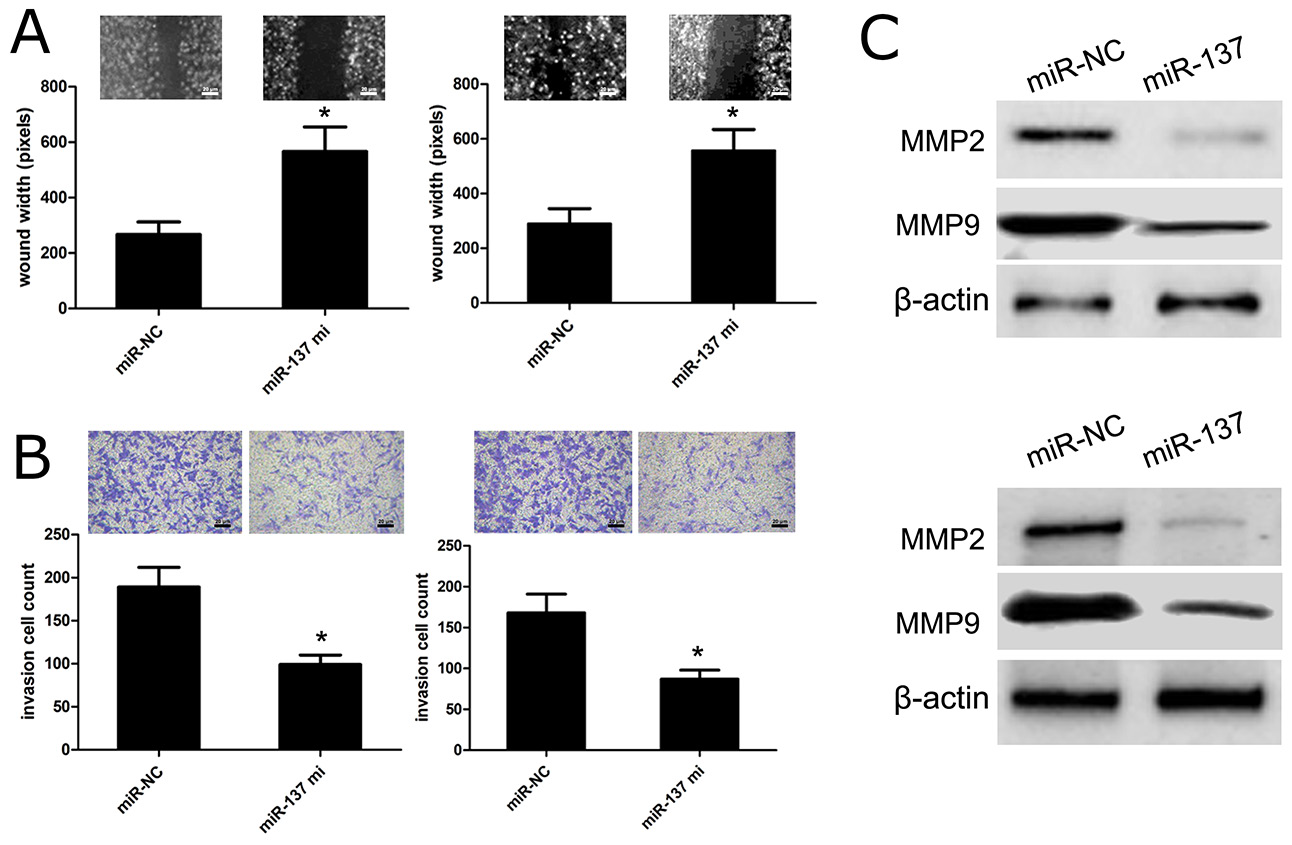
miR-137 reduces GBM cell migration and invasion **(A)** Wound healing assay performed with U87 and U251 cells over 48 h. Cells and wounds were pretreated as described above. Wound healing within the scrape line was recorded every day. Representative scrape lines are shown at day 3; dashed line indicates the margin of the scratch at day 1. **(B)** Representative fields (×10 magnification) showing invasive cells after 24 hour culture in Matrigel invasion chambers. Panels show U87 and U251 cell invasion after transfection with miR-137 mimics or miR-NC. Quantitative analysis of cell invasion experiments demonstrates that miR-137 decreases U87 and U251 cell invasion compared to miR-NC (^*^p<0.01, vs. miR-NC control). **(C)** Relative MMP2/9 expressions in U87 and U251 cells were measured after the cells were transfected with miR-137 mimics or miR-NC using western blot. Results shown are the mean ± SD of 3 repeated independent experiments. Bar=20 μm, ^*^p<0.01, compared with miR-NC, one-way ANOVA.

To figure out the effect of miR-137 on cell migration and invasion, we carried out the wound healing and transwell assays using U87 and U251 cells transfected with miR-137 mimics or with miR-NC. The wound healing analysis showed that miR-137 mimics had the capacity to inhibit U87 or U251 cell migration as compared with miR-NC group (p<0.01) (Figure 4A). In addition, the transwell assay revealed that miR-137 mimics could affect U87 and U251 cell invasion as compared with miR-NC (p<0.01) (Figure 4B). Besides, we applied western blot to investigate the change of cell invasion-related molecules, and observed that the expression of MMP2 and MMP9 protein in U87 and U251 cells with miR-137 mimics was obviously reduced, while their expression levels were increased in U87 and U251 cells with miR-NC (p<0.01) (Figure 4C). These results indicated that miR-137 exerts the inhibitory effects on GBM cell migration and invasion.

### The 3′-UTR of CXCL12 is a direct target of miR-137

**Figure 5:**
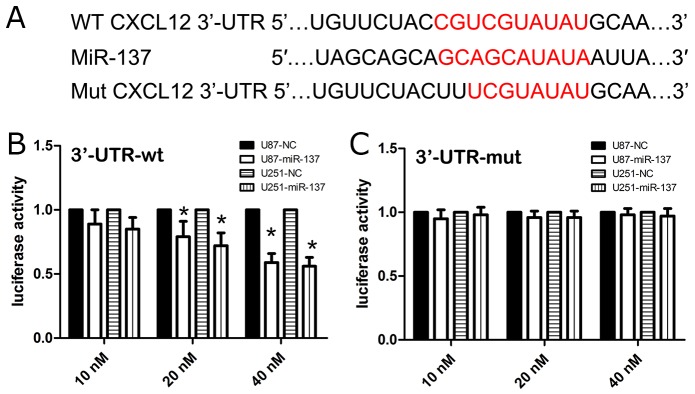
CXCL12 is a candidate target of miR-137 **(A)** The WT and Mut of 3′UTR of CXCL12 mRNA contains the binding sequences of miR-137. The miR-137 mimic inhibited the luciferase activity controlled by wild-type CXCL12-3’-UTR **(B)** but did not affect the luciferase activity controlled by mutant CXCL12-3’-UTR **(C)** in U87 and U251 cells. Results shown are the mean ± SD of 3 repeated independent experiments. ^*^p<0.01, compared with miR-NC, one-way ANOVA.

In the present study, firstly, we used three miRNA databases to predict common and putative miR-137-binding sequences located in the 3′-UTR of CXCL12 mRNA (Figure 5A). To identify whether the 3′-UTR of CXCL12 mRNA was a direct target of miR-137, we inserted a 3′-UTR (wt/mut) sequence of CXCL12 mRNA into a luciferase reporter vector, and then we detected the luciferase density. Besides, the expression level of CXCL12 protein was also detected in U87 cell lines by western blot. We observed that miR-137 mimics decreased the luciferase intensity of U87 cells transfected with CXCL12-3’UTR-wt in a dose-dependent fashion (Figure 5B), while miR-137 mimics did not altere the luciferase activity of U87 cells transfected with CXCL12-3’UTR-mut (Figure 5C). Furthermore, the expression of CXCL12 protein was reduced in the miR-137 mimics and 3′-UTR-wt-co-transfected U87 cell lines as compared with the miR-137 mimics and 3′-UTR-mut-co-transfected U87 cell lines These results suggested that the 3′-UTR of CXCL12 is a direct target of miR-137.

### CXCL12 over-expression attenuates miR-137-mediated inhibition

**Figure 6:**
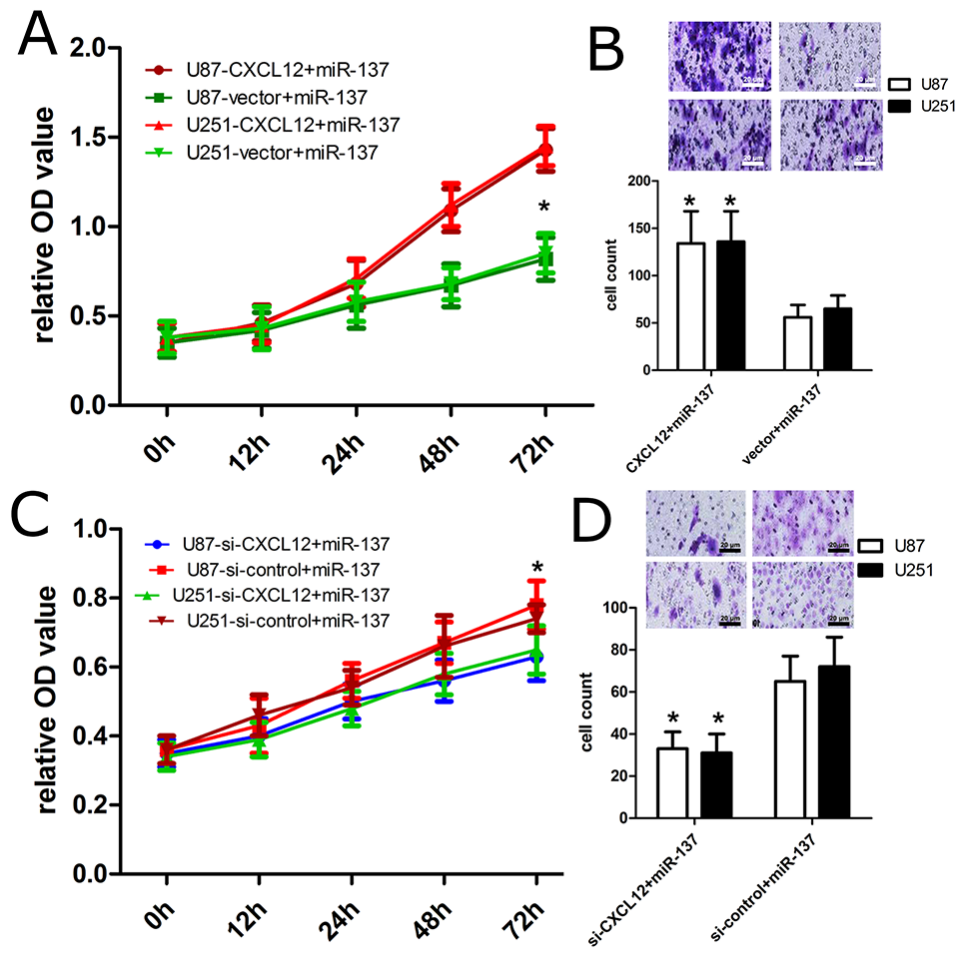
CXCL12 affects the effect of miR-137 on cell proliferation and invasion **(A)** The proliferation capacity of miR-137-overexpressing U87 and U251 cells was partially improved when cells were transfected with CXCL12 plasmids in comparison with miR-NC. **(B)** The invasion capacity of miR-137-overexpressing U87 and U251 cells were effectively improved when cells were transfected with CXCL12 plasmids. ^*^p<0.01, vs. control. **(C)** The proliferation capacity of miR-137-overexpressing U87 and U251 cells was partially inhibited when cells were transfected with si-CXCL12 compared with si-control. **(D)** The invasion capacity of miR-137-overexpressing U87 and U251 cells were effectively improved when cells were transfected with si-CXCL12. Bar=20 μm, ^*^ *p*<0. 01, vs. si-control.

In order to identify the effect of CXCL12 on miR-137-mediated the development of GBM, the pcDNA3.1(+)-CXCL12 plasmids were co-transfected into U87 and U251 cells with miR-137 mimics. In the present study, CXCL12 plasmids effectively promoted the expression of CXCL12 proteins. CCK-8 assay analysis revealed that overexpression of CXCL12 facilitated cell proliferation of U87 and U251 cells (Figure 6A). In addition, our transwell analysis further showed that CXCL12 plasmids in U87 and U251 cells with miR-137 mimics promoted U87 and U251 cell invasion as compared with their controls (Figure 6B).

### Inhibition of CXCL12 expression enhances miR-137-mediated inhibition

In order to identify the effect of CXCL12 expression inhibition on miR-137-mediated the development of GBM, the CXCL12 siRNAs were co-transfected into U87 and U251 cells with miR-137 mimics. In the present study, CXCL12 siRNAs effectively inhibited the expression of CXCL12 proteins. CCK-8 assay analysis revealed that low expression of CXCL12 further inhibited U87 and U251 cell proliferation (Figure 6C). In addition, our transwell analysis further showed that CXCL12 siRNAs in U87 and U251 cells with miR-137 mimics inhibited U87 and U251 cell invasion as compared with their controls (Figure 6D).

### MiR-137 affects engrafted tumor growth *in vivo*

**Figure 7:**
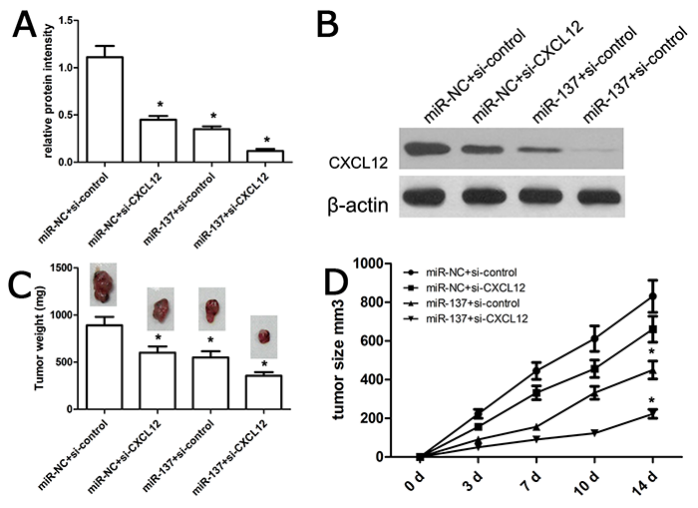
miR-137 affected the growth of U87-engrafted tumor **(A, B)** The expression of CXCL12 protein was detected using western blot assay. **(C, D)** Tumor size was measured every week using a caliper. At 14 days after cell injection, animals were sacrificed and xenograft tumors were excised and weighted. We identified that miR-137 mimics could repress the U87-engrafted tumor growth and volume as compared with miR-NC-transfected U87-engrafted tumors. Results shown are the mean ± SD of 3 repeated independent experiments. ^*^p<0.01, compared with miR-NC, one-way ANOVA.

As mentioned above, in-vitro assay demonstrated that miR-137 expression affected GBM progression. Next, we carried out in-vivo tumorigenesis assay using nude mice, and all nude mice with intra-tumor injection of miR-137 mimics or miR-NC survived normally after two weeks. We demonstrated no toxic effects of miR-137 on nude mice. Subsequently all engrafted tumors were resected, weighted and assayed. We found that miR-137 mimics or si-CXCL12 obviously inhibited the expression of CXCL12 than miR-NC or si-control group respectively (Figure 7A). In addition, miR-137 mimics and si-CXCL12 synergically inhibited the expression of CXCL12 (Figure 7B). Tumor weight analysis showed that the weight value of U87-engrafted tumor with miR-137 mimics was significantly lower than their controls (Figure 7C). Tumor volume analysis showed that the volume value of U87-engrafted tumor with miR-137 mimics got significantly slower than their controls (Figure 7D), indicating that miR-137 mimics repressed U87-engrafted tumor growth.

## DISCUSSION

Emerging evidence has shown that the CXCL12/CXCR4 signaling pathway plays an important role in many kinds of biological processes including stem cell migration and homing, inflammation developmetn, and immunoregulation [11–14]. At the same time, CXCL12 signaling pathway was also involved into the development of tumors, such as cell growth and metastasis [15–17]. Importanty, the expression of CXCL12 has been identified to be involved into the initiation and progression of human GBM. In addition, it has been reported that the miRNAs were often down-regulated, and could induce oncogenesis by silencing tumor suppressor genes [18]. Accompanied with the development of GBM, specific aberrant miRNAs have been identified, and their expressions were closely related with cell proliferation, migration and invasion. However, the expression and the role of miR-137 in GBM have not been clearly reported till now.

Our study firstly showed that the expression of miR-137 was reduced in tumor tissues than that in paired non-tumor brain tissues. However, the expression of mRNA and protein of CXCL12 had much higher expression in tumor tissues than those in paired normal tissues. These results were consistent with some previous reports, indicating that miR-137 is involved into the development of human GBM [18]. *In vitro* data further indicated that miR-137 expression plays a suppressive role in tumor cell proliferation, migration and invasion. Subsequently, we used bioinformatics analysis and cell transfection to demonstrate the tumor-suppressing effects of miR-137 were due to down-regulation of CXCL12. This is the first study to explore the post-transcriptional regulation of CXCL12 by miR-137 in human GBM.

Recent reports indicated that EGF and its receptor EGFR were increased in some tumors, such as gastric cancer, and GBM, which further induced tumorigenesis, and stimulated cell proliferation and migration [19–21]. In addition, matrix metalloproteinases are involved into cell invasion, metastases, and angiogenesi, and can cause dynamic changes of biological behaviors [22–25]. In the present work, our study showed that U87 and U251 cells transfected with miR-137 mimics indeed decreased the expression of EGFR, MMP2 and MMP9 proteins than control, suggesting that miR-137 reduced GBM cell proliferation and invasion. These findings suggested that miR-137 expression exerts the inhibitory effects on GBM cell migration and invasion.

In conclusion, our work demonstrated that miR-137 serves as a tumor suppressor by inhibition of CXCL12 in human GBM. Thus, miR-137-CXCL12 can be recommended as a useful and effective target for treatment of GBM.

## MATERIALS AND METHODS

### Ethics Statement

The present study was approved by the Ethics Committee of Zhejiang University and Jinzhou Medical University. Patients enrolled in this study signed written informed consent. All procedures were subjected to the Declaration of Helsinki.

### Patients and tissues

50 cases of GBM specimens were obtained from patients during surgery at the First Affiliated Hospital of Zhejiang University. A portion of the tumor tissues were saved and made into paraffin sections for histopathologic diagnosis in strict accordance with World Health Organization (WHO) criteria by two established neuropathologists, with differences resolved by careful discussion. And the remaining tissue was snap-frozen in liquid nitrogen then stored at -80°C for RNA extraction and other biological molecular experiments. Before the RNA extraction from frozen tissues, the adjacent tumor tissues were subjected to frozen sections and reviewed by a pathologist to ensure that a minimum of 80% tumor cells were included in the sample. For GBM patients, none of them had received chemotherapy or radiotherapy prior to surgery, and all patients were well followed up. Patients, who died of diseases not directly related to their gliomas or due to unexpected events, were excluded from this study.

### Cell culture

As cell culture, primary normal human astrocytes (NHA) were purchased from the Sciencell Research Laboratories (Carlsbad, CA) and cultured under the conditions as instructed by the manufacturer. Cell lines U87, and U251 were obtained from the KeyGEN Company (China) and cultured in DMEM, 10% fetal bovine serum (Gibco, Grand Island, NY, USA) and 1% penicillin-streptomycin (Gibco, Grand Island, NY, USA) at 37°C in a humidified atmosphere under 5% CO2. The medium was replaced every 3 days.

### Reverse transcription and Taqman real-time polymerase chain reaction (qPCR)

Total RNA was reverse transcribed into cDNA using the TaqMan Reverse Transcription kit (Applied Biosystems, Carlsbad, CA, USA) in a 15 μL reaction mix containing 100 mM dNTPs, 10X Reverse Transcriptase Buffer, 20 U/μL RNase Inhibitor, 50 U/μl MultiScribe Reverse Transcriptase, and 5X RT primer. The mixture was incubated at 16°C for 30 minutes, 42°C for 30 minutes, and 85°C for 5 minutes. The resulting first-strand complementary DNA (cDNA) was used as template for TaqMan-based Real-Time PCR as follows: 1 μL of cDNA was added to TaqMan Universal PCR Master Mix II (Applied Biosystems, Carlsbad, CA, USA) containing a TaqMan Gene Expression Assay (20X) for each primer. PCR was performed using a StepOne plus (Applied Biosystems) real-time PCR instrument with the following thermal settings: one cycle of 2 minutes at 50°C, one cycle of 10 minutes at 95°C, and 40 cycles of 15 seconds at 95°C and 1 minute at 60°C. The threshold cycle (Ct) was used to calculate relative miRNA expression levels (2-ΔΔCt) [23, 24]. RNU48 was used as an endogenous control. For each miRNA, ΔCt was calculated as the Ct of each sample minus the Ct of RNU48, and the ΔΔCt was calculated as the ΔCt of each sample minus the mean ΔCt of the control group. Values are expressed as “Relative Expression”.

The PCR primers sets used here for miR-137 was designed as follows: miR-137 forward primer: 5’-GCGCGC TTATTGCTTAAGAATAC-3’, and reverse primer: 5’-GTGCAGGGTCCGAGGT-3’. U6 was used as an internal control and amplified with forward primer: 5’-GCTTCGGCAGCACATATACTAAAAT-3’, and reverse primer: 5’-CGCTTCACGAATTTGCGT GTCAT-3’. human CXCL12 forward primer: 5’-CTCCTGGGGATGTGTAATGG-3’ and reverse 5’-GCCTCCATGGCATACATAGG-3’; human GAPDH forward primer: 5’-GGGCATCCTGGGCTACACTG-3’ and reverse 5’-GAGGTCCACCACCCTGTTGC-3’.

### Transient transfection of miR-137 oligonucleotides

Cells were transiently transfected with 50 nmol of the miR-137 mimic with Lipofectamine 2000 (Invitrogen) according to the manufacturer's recommendation. The antisense oligonucleotides used in these studies were the miR-137 primer as mentioned above; the miRNA mimic-negative control (NC mimic): 5’-UUCUCCGAACGUGUCACGUTT-3’ and 5’-ACGUGACACGUUCGGAGAATT-3’. All miRNA oligonucleotides were purchased from Genepharma (Shanghai, China).

### Western blot analysis

For the protein analysis, the cells were harvested at 12~24 h following different treatments, as described above, and washed with cold PBS and then incubated in ice-cold RIPA buffer. Cell lysates were sonicated for 30 s on ice and lysed at 4°C for 60 min. Then, the cell lysates were centrifuged at 12,000 g for 30 min at 4°C. Protein concentrations in the supernatants were determined by the BCA reagent. Total protein was separated by denaturing 8–12% SDS-polyacrylamide gel electrophoresis, which was resolved over and electrotransferred by semidry blotting (Bio-Rad Laboratories, Shanghai) onto a nitrocellulose membrane. The membrane was incubated with primary antibodies (Abcam, Cambridge, UK, 1:1000 dilution) or β-actin (Santa Cruz Biotech, Santa Cruz, CA, 1:1000 dilution) overnight at 4°C, and then with peroxidase-conjugated secondary antibody (Santa Cruz Biotech, Santa Cruz, CA, 1:1000 dilution), visualized by chemiluminescence (GE, Fairfield, CT, USA).

### Cell proliferation assay

Cells were seeded at 2,000 per well in 96-well plates and cultured after transfection. Cell proliferation was detected at the indicated time points using a CCK-8 kit (Dojindo Laboratories) following the manufacturer's instructions. All assays were performed in octuplicate and repeated at least three times.

### Wound-healing assay

Cells were seeded in 12-well plates and grown to 90% confluence. Cells were transfected with or without NC miRNAs or miR-137 mimics. After 36 h of transfection, cells were serum starved overnight and a linear wound was created using a pipette tip. Wound closure was monitored using live cell imaging microscopy at an interval of 30 min for 24–48 h. Wound size was then measured randomly at three sites perpendicular to the wound.

### Transwell invasion assays

As for transwell assay, we seeded cells on the upper chamber of each insert, and then, 500 μl of DMEM (10% FBS) was added to a 24-well plate for 12 hour incubation at 37°C, the cells on the lower layer were collected, and fixed with a 0.1% crystal violet. As for invasion assay, transwell chambers were uniformly plated with 60 μl Matrigel diluted with DMEM, and then incubated for 4 h at 37°C, and then the same procedures with migration assay were conducted.

### Luciferase reporter assay

A dual-luciferase reporter vector was used to generate the luciferase constructs. The target genes of miR-137 were selected based on target scan algorithms [microRNA.org (http://www.microrna.org/microrna/home.do) Microcosm (http://www.ebi.ac.uk/ enright-srv/microcosm/htdocs/targets/) and TargetScan (http://www.targetscan.org/)]. For 3’UTR luciferase assay, the putative binding sites of miR-137 and its homologous mutation sites in the 3’-UTR region of CXCL12 mRNA were amplified and cloned into pGL3-contral luciferase reporter plasmid (Invitrogen, Carlsbad, CA). The pRL vector constitutively expressing Renilla luciferase was used to normalize for trasfection efficiency. Luciferase activity was measured using the Dual-Luciferase Reporter Assay System (Promega, Madison, USA) after transfection at 48 h. Data are presented as the mean value ± standard deviations (SD) for triplicate experiments.

### *In vivo* tumorigenesis assay

The experiments involving animals were performed in accordance with the institutional guidelines for animal care and were approved by the University Committee for the Use and Care of Animals. Male BALB/c nude mice (4 weeks old and weighing about 20 g) were purchased from Shanghai Laboratory Animal Center (Shanghai, China). miR-137-overexpressing or control cells (5 × 10^6^ per mouse, 3 mice per group) were subcutaneously injected into the right flanks of mice. Tumor size was measured every week using a caliper. At 14 days after cell injection, animals were sacrificed and xenograft tumors were excised and weighted. We detected the tumor length, width and weight every 3 days. All mice in this study were euthanized by way of cervical traction at 14 days following tumor cell inoculation.

### Statistical analysis

Significance was determined using the one-way ANOVA test on the mean values of three different experiments. Significance was determined using the mean ± SD and was analyzed by 2-tailed Student's t-tests using the Statistical Program for Social Sciences 13.0 software (SPSS Corp., Shanghai, China). P<0.05 was used as the cutoff for statistically significant differences. In the Western blotting analysis, the corresponding strips used to estimate the value of the relative protein content were captured photographically through a Bio-Rad image analysis system with Image-Pro software analysis.
